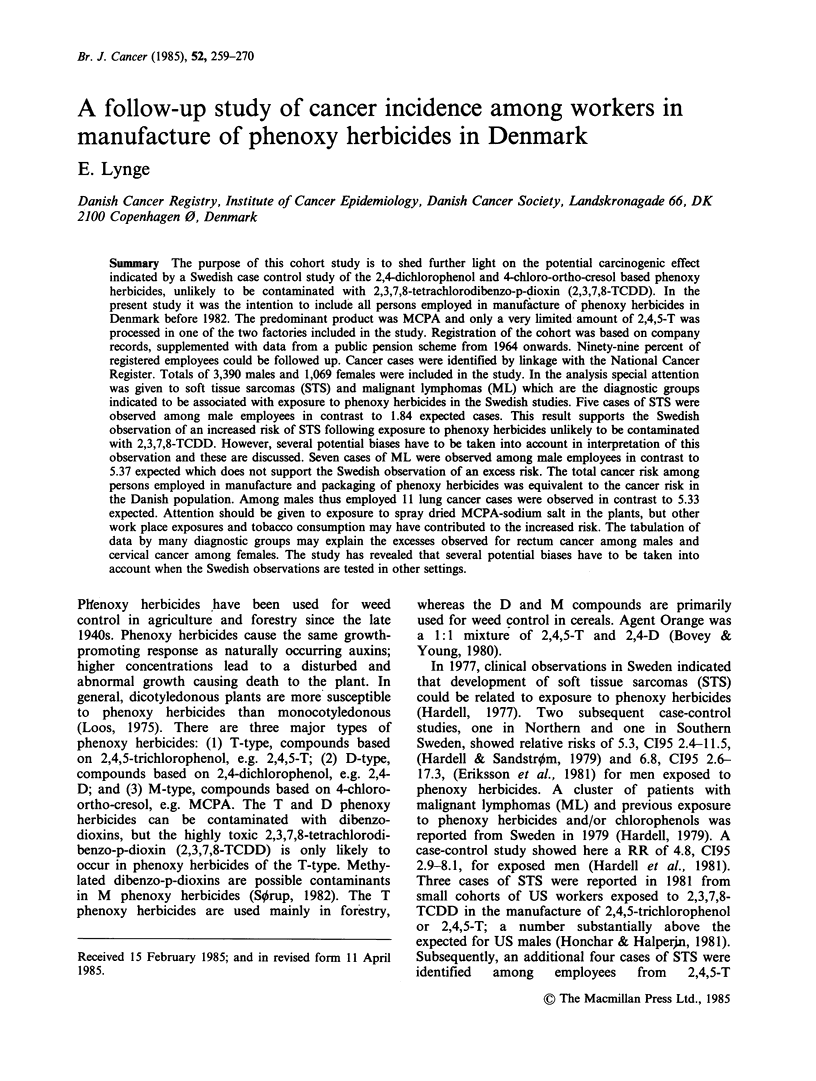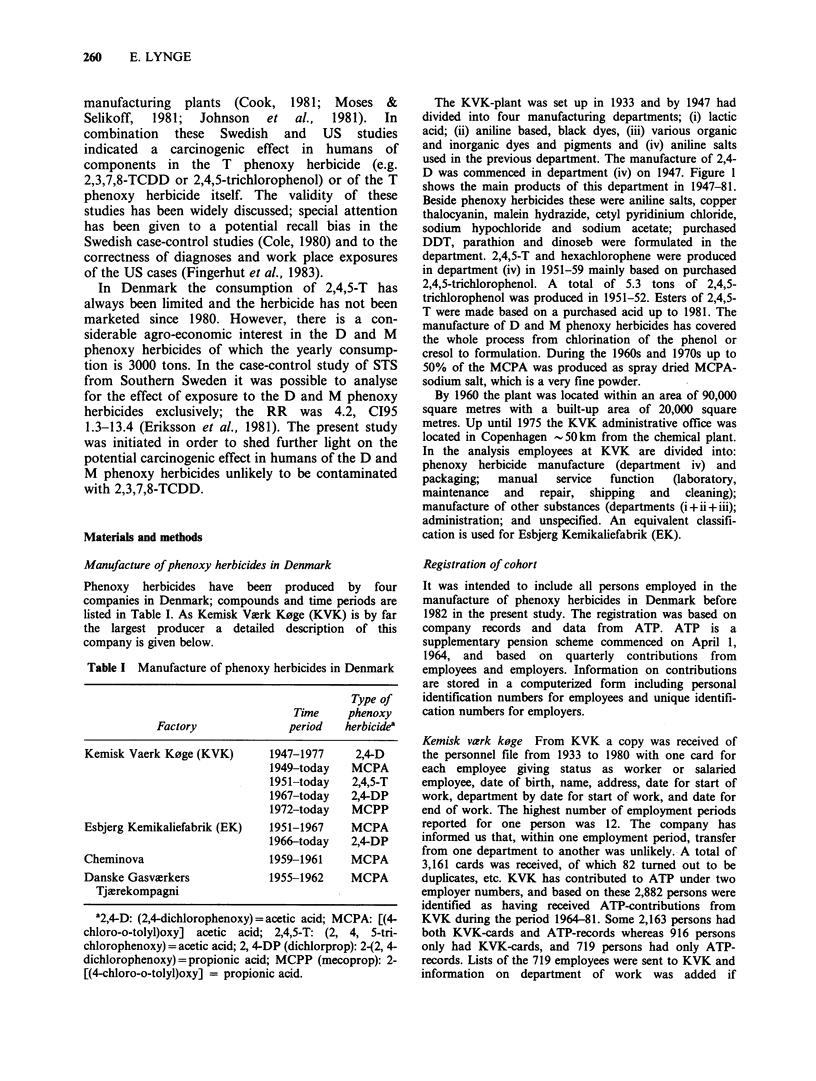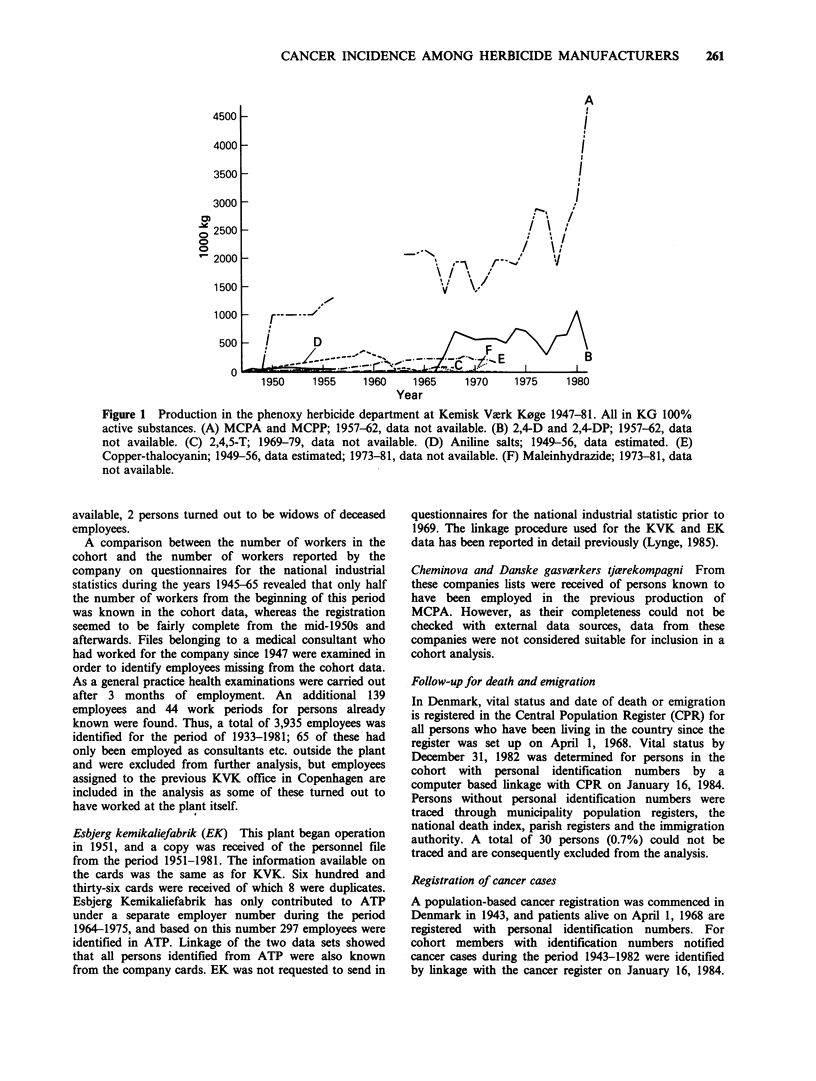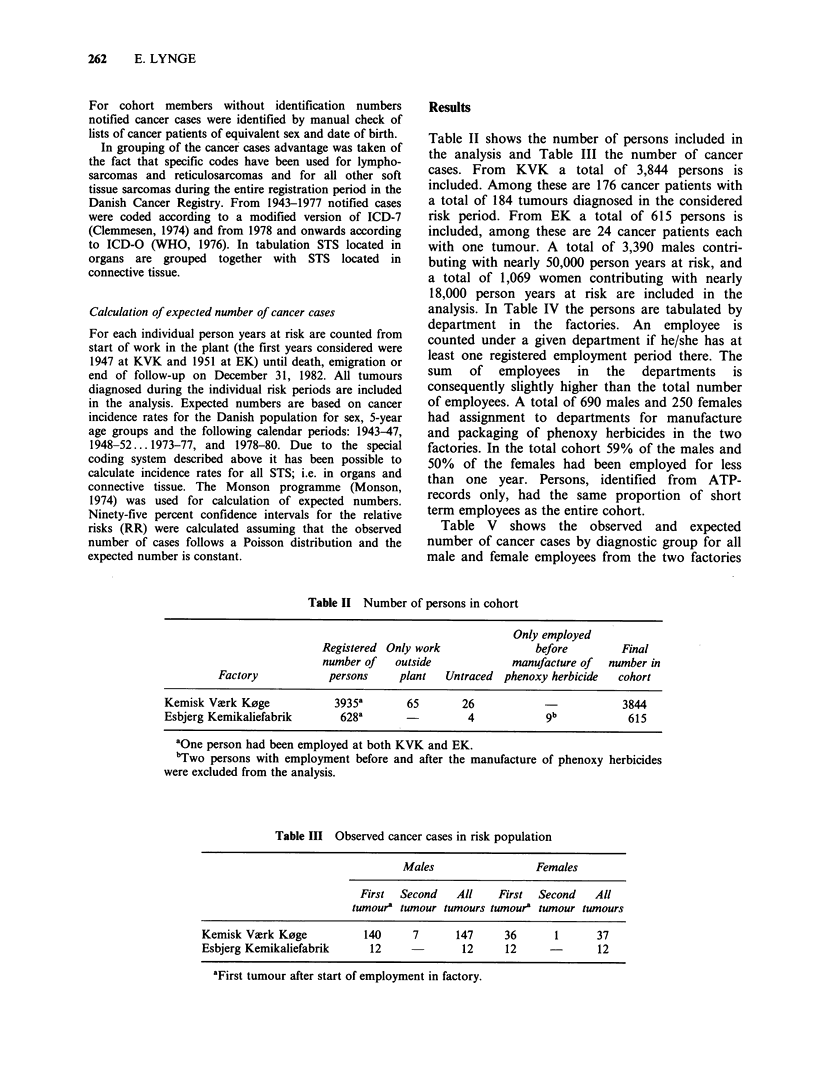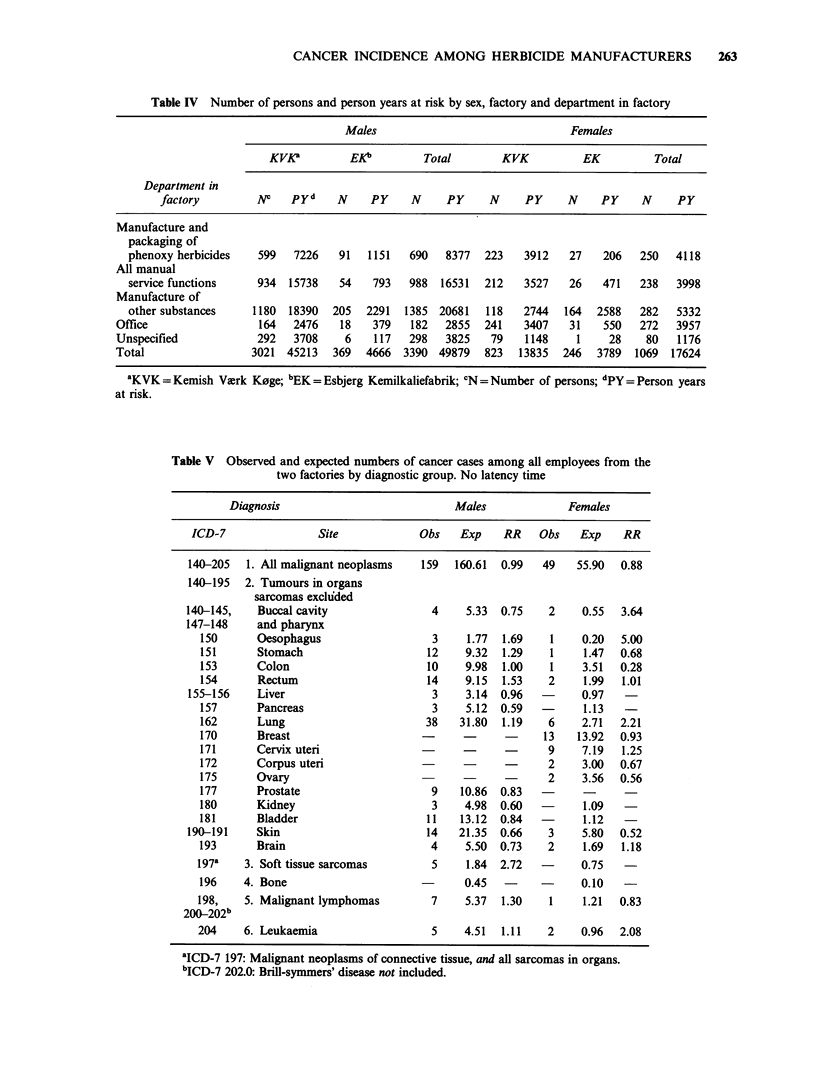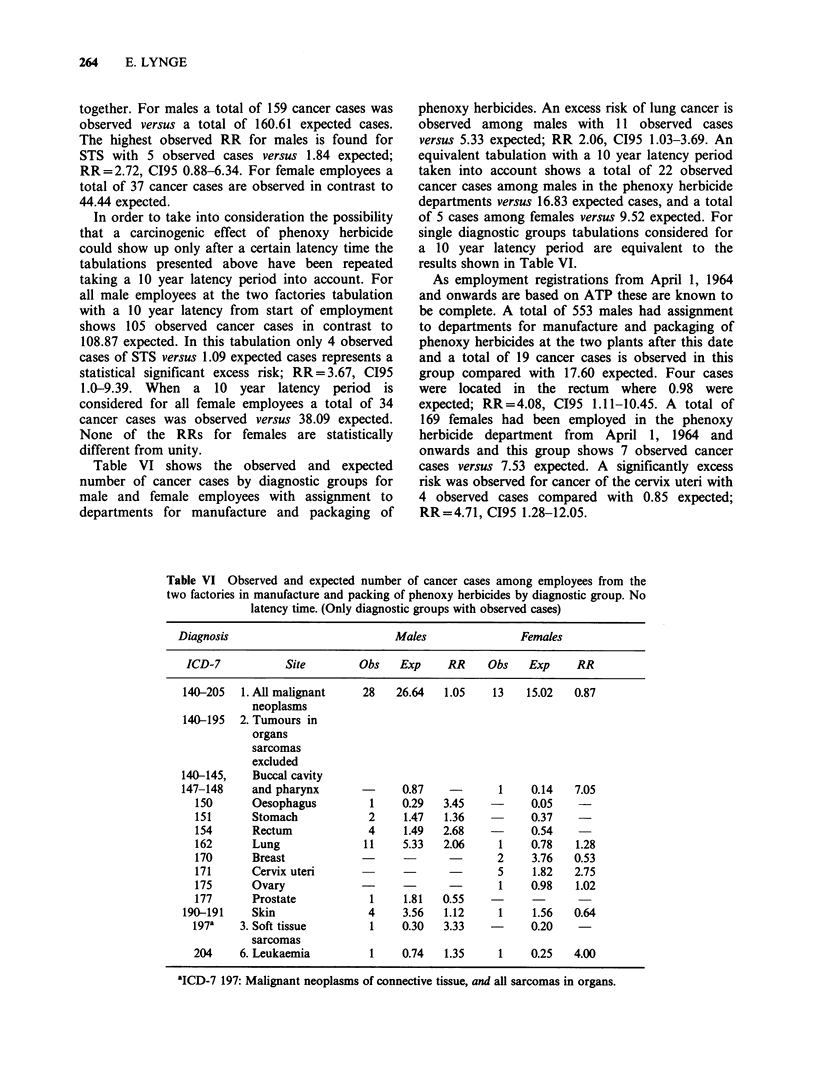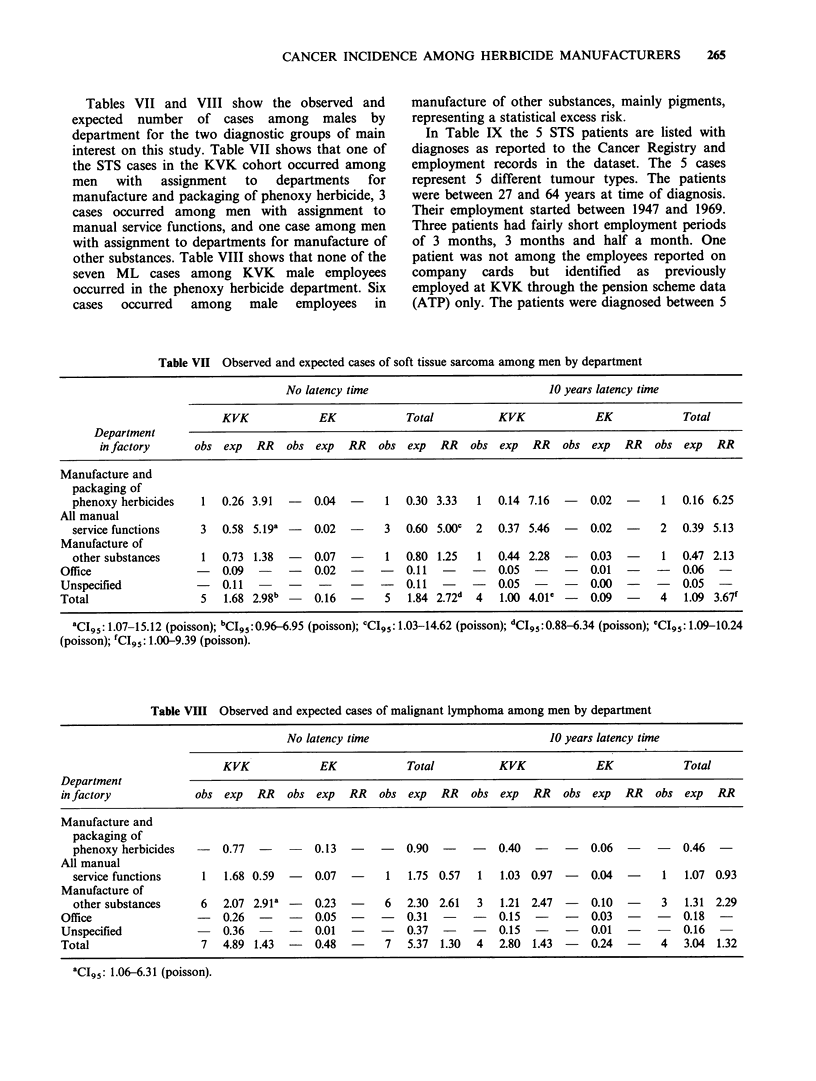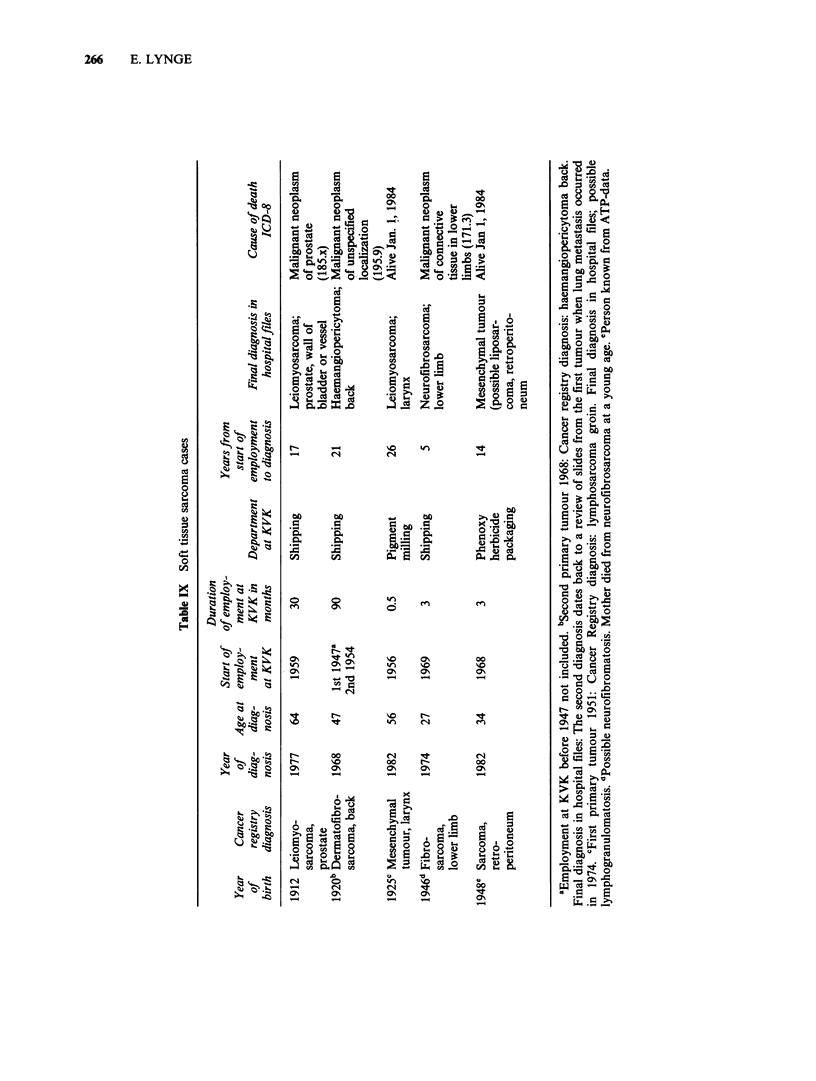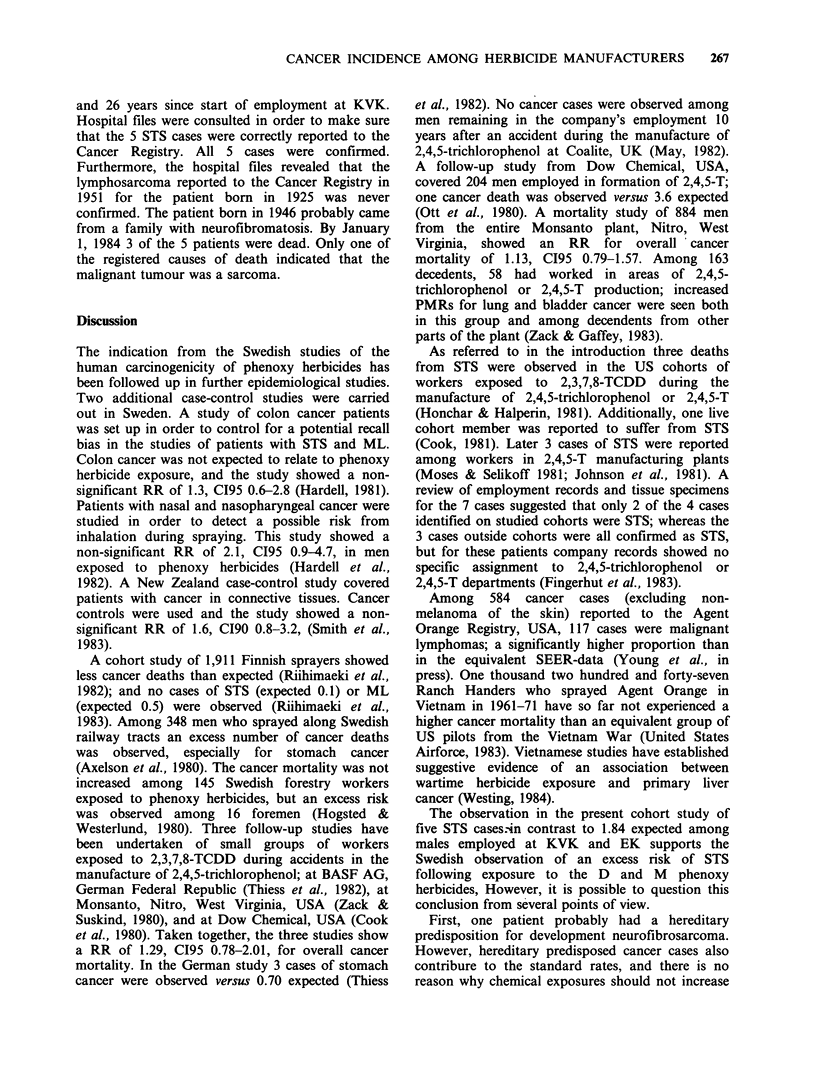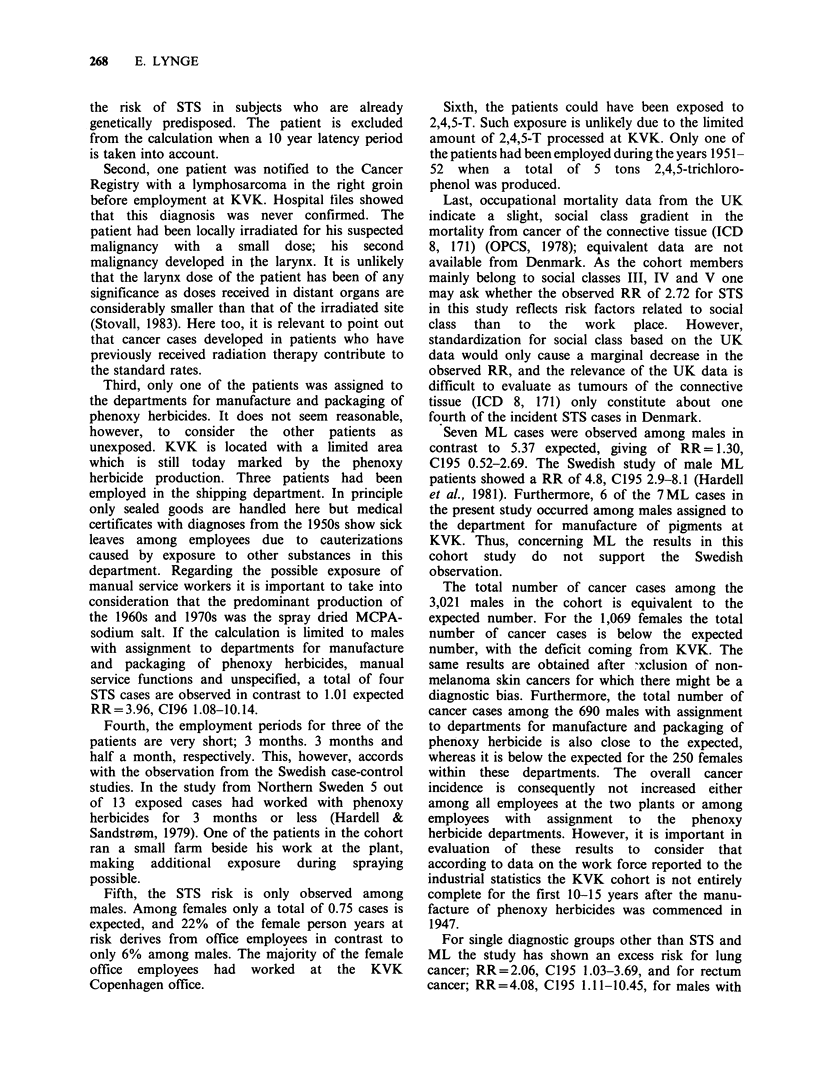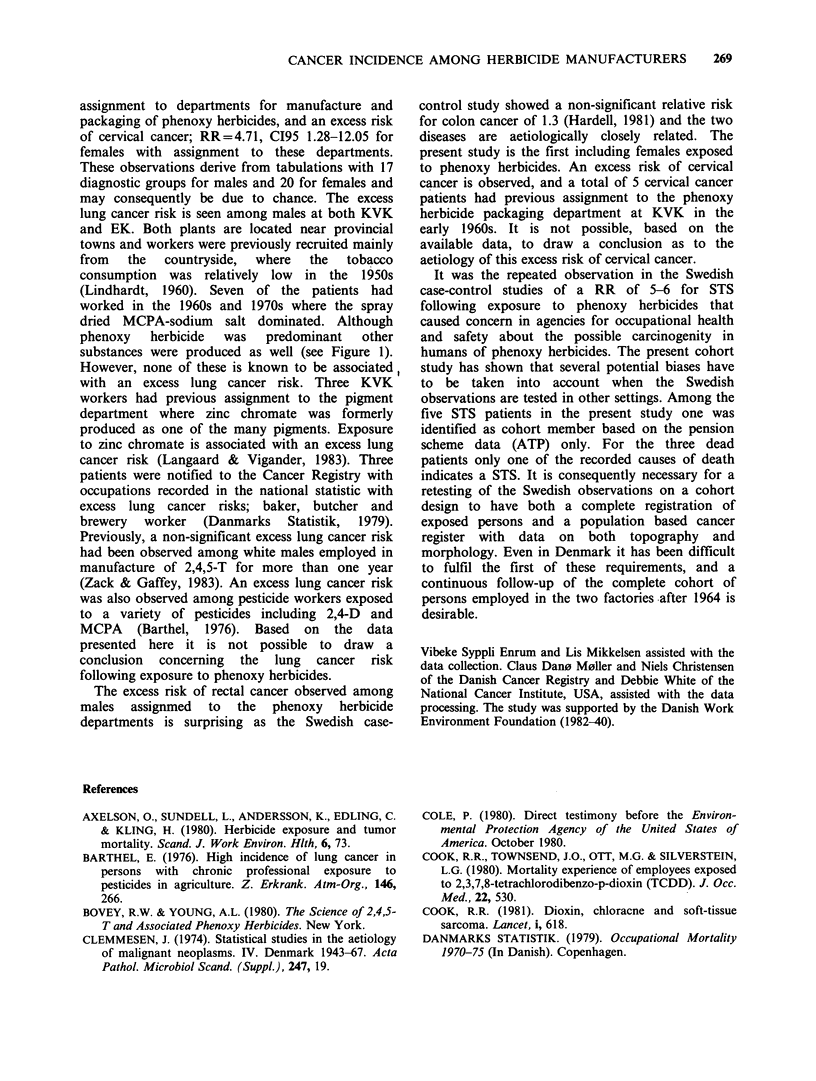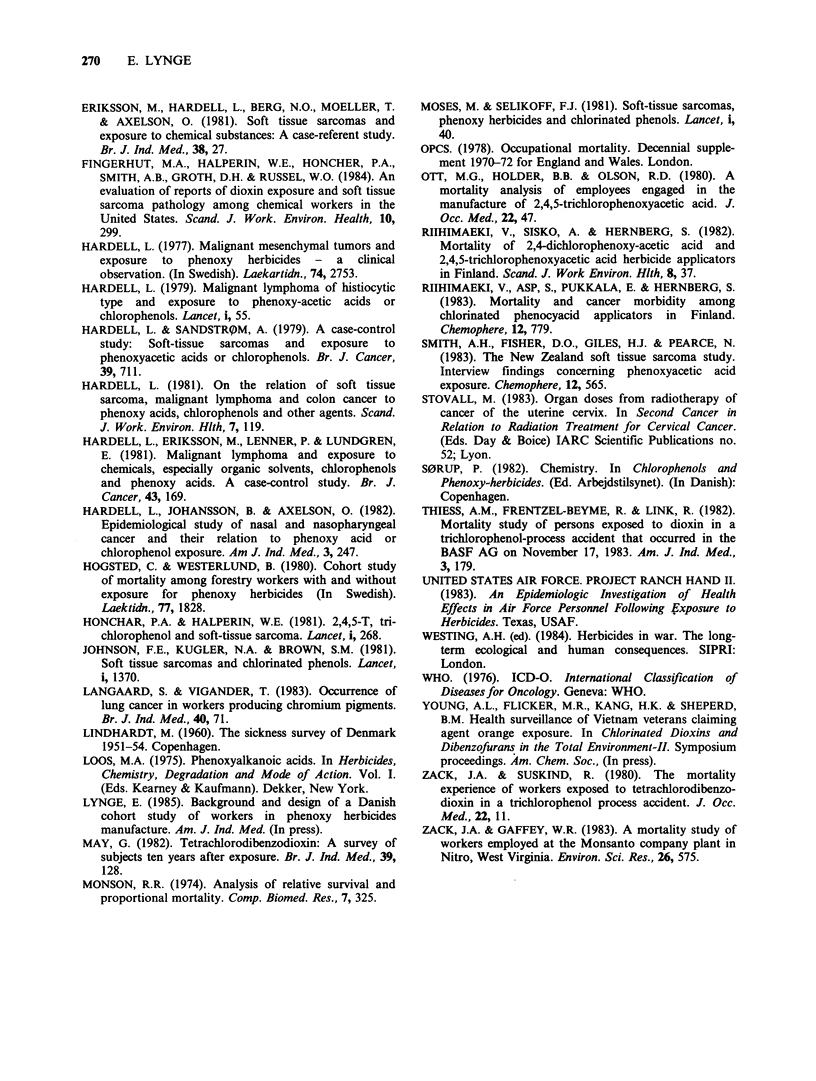# A follow-up study of cancer incidence among workers in manufacture of phenoxy herbicides in Denmark.

**DOI:** 10.1038/bjc.1985.186

**Published:** 1985-08

**Authors:** E. Lynge

## Abstract

The purpose of this cohort study is to shed further light on the potential carcinogenic effect indicated by a Swedish case control study of the 2,4-dichlorophenol and 4-chloro-ortho-cresol based phenoxy herbicides, unlikely to be contaminated with 2,3,7,8-tetrachlorodibenzo-p-dioxin (2,3,7,8-TCDD). In the present study it was the intention to include all persons employed in manufacture of phenoxy herbicides in Denmark before 1982. The predominant product was MCPA and only a very limited amount of 2,4,5-T was processed in one of the two factories included in the study. Registration of the cohort was based on company records, supplemented with data from a public pension scheme from 1964 onwards. Ninety-nine percent of registered employees could be followed up. Cancer cases were identified by linkage with the National Cancer Register. Totals of 3,390 males and 1,069 females were included in the study. In the analysis special attention was given to soft tissue sarcomas (STS) and malignant lymphomas (ML) which are the diagnostic groups indicated to be associated with exposure to phenoxy herbicides in the Swedish studies. Five cases of STS were observed among male employees in contrast to 1.84 expected cases. This result supports the Swedish observation of an increased risk of STS following exposure to phenoxy herbicides unlikely to be contaminated with 2,3,7,8-TCDD. However, several potential biases have to be taken into account in interpretation of this observation and these are discussed. Seven cases of ML were observed among male employees in contrast to 5.37 expected which does not support the Swedish observation of an excess risk. The total cancer risk among persons employed in manufacture and packaging of phenoxy herbicides was equivalent to the cancer risk in the Danish population. Among males thus employed 11 lung cancer cases were observed in contrast to 5.33 expected. Attention should be given to exposure to spray dried MCPA-sodium salt in the plants, but other work place exposures and tobacco consumption may have contributed to the increased risk. The tabulation of data by many diagnostic groups may explain the excesses observed for rectum cancer among males and cervical cancer among females. The study has revealed that several potential biases have to be taken into account when the Swedish observations are tested in other settings.


Br. J. Cancer (1985), 52, 259–270

### A follow-up study of cancer incidence among workers in manufacture of phenoxy herbicides in Denmark

E. Lynge

*Danish Cancer Registry, Institute of Cancer Epidemiology, Danish Cancer Society, Landskronagade 66, DK 2100 Copenhagen Ø, Denmark*

**Summary** The purpose of this cohort study is to shed further light on the potential carcinogenic effect indicated by a Swedish case control study of the 2,4-dichlorophenol and 4-chloro-ortho-cresol based phenoxy herbicides, unlikely to be contaminated with 2,3,7,8-tetrachlorodibenzo-p-dioxin (2,3,7,8-TCDD). In the present study it was the intention to include all persons employed in manufacture of phenoxy herbicides in Denmark before 1982. The predominant product was MCPA and only a very limited amount of 2,4,5-T was processed in one of the two factories included in the study. Registration of the cohort was based on company records, supplemented with data from a public pension scheme from 1964 onwards. Ninety-nine percent of registered employees could be followed up. Cancer cases were identified by linkage with the National Cancer Register. Totals of 3,390 males and 1,069 females were included in the study. In the analysis special attention was given to soft tissue sarcomas (STS) and malignant lymphomas (ML) which are the diagnostic groups indicated to be associated with exposure to phenoxy herbicides in the Swedish studies. Five cases of STS were observed among male employees in contrast to 1.84 expected cases. This result supports the Swedish observation of an increased risk of STS following exposure to phenoxy herbicides unlikely to be contaminated with 2,3,7,8-TCDD. However, several potential biases have to be taken into account in interpretation of this observation and these are discussed. Seven cases of ML were observed among male employees in contrast to 5.37 expected which does not support the Swedish observation of an excess risk. The total cancer risk among persons employed in manufacture and packaging of phenoxy herbicides was equivalent to the cancer risk in the Danish population. Among males thus employed 11 lung cancer cases were observed in contrast to 5.33 expected. Attention should be given to exposure to spray dried MCPA-sodium salt in the plants, but other work place exposures and tobacco consumption may have contributed to the increased risk. The tabulation of data by many diagnostic groups may explain the excesses observed for rectum cancer among males and cervical cancer among females. The study has revealed that several potential biases have to be taken into account when the Swedish observations are tested in other settings.

Phenoxy herbicides have been used for weed control in agriculture and forestry since the late 1940s. Phenoxy herbicides cause the same growth-promoting response as naturally occurring auxins; higher concentrations lead to a disturbed and abnormal growth causing death to the plant. In general, dicotyledonous plants are more susceptible to phenoxy herbicides than monocotyledonous (Loos, 1975). There are three major types of phenoxy herbicides: (1) T-type, compounds based on 2,4,5-trichlorophenol, e.g. 2,4,5-T; (2) D-type, compounds based on 2,4-dichlorophenol, e.g. 2,4-D; and (3) M-type, compounds based on 4-chloro-ortho-cresol, e.g. MCPA. The T and D phenoxy herbicides can be contaminated with dibenzo-dioxins, but the highly toxic 2,3,7,8-tetrachlorodibenzo-p-dioxin (2,3,7,8-TCDD) is only likely to occur in phenoxy herbicides of the T-type. Methylated dibenzo-p-dioxins are possible contaminants in M phenoxy herbicides (Søgrup, 1982). The T phenoxy herbicides are used mainly in forestry, whereas the D and M compounds are primarily used for weed control in cereals. Agent Orange was a 1:1 mixture of 2,4,5-T and 2,4-D (Bovey & Young, 1980).

In 1977, clinical observations in Sweden indicated that development of soft tissue sarcomas (STS) could be related to exposure to phenoxy herbicides (Hardell, 1977). Two subsequent case-control studies, one in Northern and one in Southern Sweden, showed relative risks of 5.3, CI95 2.4–11.5, (Hardell & Sandstrøm, 1979) and 6.8, CI95 2.6–17.3, (Eriksson *et al.*, 1981) for men exposed to phenoxy herbicides. A cluster of patients with malignant lymphomas (ML) and previous exposure to phenoxy herbicides and/or chlorophenols was reported from Sweden in 1979 (Hardell, 1979). A case-control study showed here a RR of 4.8, CI95 2.9–8.1, for exposed men (Hardell *et al.*, 1981). Three cases of STS were reported in 1981 from small cohorts of US workers exposed to 2,3,7,8-TCDD in the manufacture of 2,4,5-trichlorophenol or 2,4,5-T; a number substantially above the expected for US males (Honchar & Halperin, 1981). Subsequently, an additional four cases of STS were identified among employees from 2,4,5-T

Received 15 February 1985; and in revised form 11 April 1985.

© The Macmillan Press Ltd., 1985

manufacturing plants (Cook, 1981; Moses & Selikoff, 1981; Johnson *et al.*, 1981). In combination these Swedish and US studies indicated a carcinogenic effect in humans of components in the T phenoxy herbicide (e.g. 2,3,7,8-TCDD or 2,4,5-trichlorophenol) or of the T phenoxy herbicide itself. The validity of these studies has been widely discussed; special attention has been given to a potential recall bias in the Swedish case-control studies (Cole, 1980) and to the correctness of diagnoses and work place exposures of the US cases (Fingerhut *et al.*, 1983).

In Denmark the consumption of 2,4,5-T has always been limited and the herbicide has not been marketed since 1980. However, there is a considerable agro-economic interest in the D and M phenoxy herbicides of which the yearly consumption is 3000 tons. In the case-control study of STS from Southern Sweden it was possible to analyse for the effect of exposure to the D and M phenoxy herbicides exclusively; the RR was 4.2, CI95 1.3–13.4 (Eriksson *et al.*, 1981). The present study was initiated in order to shed further light on the potential carcinogenic effect in humans of the D and M phenoxy herbicides unlikely to be contaminated with 2,3,7,8-TCDD.

### Materials and methods

### *Manufacture of phenoxy herbicides in Denmark*

Phenoxy herbicides have been produced by four companies in Denmark; compounds and time periods are listed in Table I. As Kemisk Værk Køge (KVK) is by far the largest producer a detailed description of this company is given below.

**Table I** Manufacture of phenoxy herbicides in Denmark

| *Factory* | *Time period* | *Type of phenoxy herbicide*[a] |
|---|---|---|
| Kemisk Vaerk Køge (KVK) | 1947–1977 | 2,4-D |
| | 1949–today | MCPA |
| | 1951–today | 2,4,5-T |
| | 1967–today | 2,4-DP |
| | 1972–today | MCPP |
| Esbjerg Kemikaliefabrik (EK) | 1951–1967 | MCPA |
| | 1966–today | 2,4-DP |
| Cheminova | 1959–1961 | MCPA |
| Danske Gasværkers Tjærekompagni | 1955–1962 | MCPA |

[a]2,4-D: (2,4-dichlorophenoxy) = acetic acid; MCPA: [(4-chloro-o-tolyl)oxy] acetic acid; 2,4,5-T: (2, 4, 5-trichlorophenoxy) = acetic acid; 2, 4-DP (dichlorprop): 2-(2, 4-dichlorophenoxy) = propionic acid; MCPP (mecoprop): 2-[(4-chloro-o-tolyl)oxy] = propionic acid.

The KVK-plant was set up in 1933 and by 1947 had divided into four manufacturing departments; (i) lactic acid; (ii) aniline based, black dyes, (iii) various organic and inorganic dyes and pigments and (iv) aniline salts used in the previous department. The manufacture of 2,4-D was commenced in department (iv) on 1947. Figure 1 shows the main products of this department in 1947–81. Beside phenoxy herbicides these were aniline salts, copper thalocyanin, malein hydrazide, cetyl pyridinium chloride, sodium hypochloride and sodium acetate; purchased DDT, parathion and dinoseb were formulated in the department. 2,4,5-T and hexachlorophene were produced in department (iv) in 1951–59 mainly based on purchased 2,4,5-trichlorophenol. A total of 5.3 tons of 2,4,5-trichlorophenol was produced in 1951–52. Esters of 2,4,5-T were made based on a purchased acid up to 1981. The manufacture of D and M phenoxy herbicides has covered the whole process from chlorination of the phenol or cresol to formulation. During the 1960s and 1970s up to 50% of the MCPA was produced as spray dried MCPA-sodium salt, which is a very fine powder.

By 1960 the plant was located within an area of 90,000 square metres with a built-up area of 20,000 square metres. Up until 1975 the KVK administrative office was located in Copenhagen ~50 km from the chemical plant. In the analysis employees at KVK are divided into: phenoxy herbicide manufacture (department iv) and packaging; manual service function (laboratory, maintenance and repair, shipping and cleaning); manufacture of other substances (departments (i + ii + iii); administration; and unspecified. An equivalent classification is used for Esbjerg Kemikaliefabrik (EK).

### *Registration of cohort*

It was intended to include all persons employed in the manufacture of phenoxy herbicides in Denmark before 1982 in the present study. The registration was based on company records and data from ATP. ATP is a supplementary pension scheme commenced on April 1, 1964, and based on quarterly contributions from employees and employers. Information on contributions are stored in a computerized form including personal identification numbers for employees and unique identification numbers for employers.

*Kemisk værk køge* From KVK a copy was received of the personnel file from 1933 to 1980 with one card for each employee giving status as worker or salaried employee, date of birth, name, address, date for start of work, department by date for start of work, and date for end of work. The highest number of employment periods reported for one person was 12. The company has informed us that, within one employment period, transfer from one department to another was unlikely. A total of 3,161 cards was received, of which 82 turned out to be duplicates, etc. KVK has contributed to ATP under two employer numbers, and based on these 2,882 persons were identified as having received ATP-contributions from KVK during the period 1964–81. Some 2,163 persons had both KVK-cards and ATP-records whereas 916 persons only had KVK-cards, and 719 persons had only ATP-records. Lists of the 719 employees were sent to KVK and information on department of work was added if

**Figure 1** Production in the phenoxy herbicide department at Kemisk Værk Køge 1947–81. All in KG 100% active substances. (A) MCPA and MCPP; 1957–62, data not available. (B) 2,4-D and 2,4-DP; 1957–62, data not available. (C) 2,4,5-T; 1969–79, data not available. (D) Aniline salts; 1949–56, data estimated. (E) Copper-thalocyanin; 1949–56, data estimated; 1973–81, data not available. (F) Maleinhydrazide; 1973–81, data not available.

available, 2 persons turned out to be widows of deceased employees.

A comparison between the number of workers in the cohort and the number of workers reported by the company on questionnaires for the national industrial statistics during the years 1945–65 revealed that only half the number of workers from the beginning of this period was known in the cohort data, whereas the registration seemed to be fairly complete from the mid-1950s and afterwards. Files belonging to a medical consultant who had worked for the company since 1947 were examined in order to identify employees missing from the cohort data. As a general practice health examinations were carried out after 3 months of employment. An additional 139 employees and 44 work periods for persons already known were found. Thus, a total of 3,935 employees was identified for the period of 1933–1981; 65 of these had only been employed as consultants etc. outside the plant and were excluded from further analysis, but employees assigned to the previous KVK office in Copenhagen are included in the analysis as some of these turned out to have worked at the plant itself.

*Esbjerg kemikaliefabrik* (*EK*) This plant began operation in 1951, and a copy was received of the personnel file from the period 1951–1981. The information available on the cards was the same as for KVK. Six hundred and thirty-six cards were received of which 8 were duplicates. Esbjerg Kemikaliefabrik has only contributed to ATP under a separate employer number during the period 1964–1975, and based on this number 297 employees were identified in ATP. Linkage of the two data sets showed that all persons identified from ATP were also known from the company cards. EK was not requested to send in questionnaires for the national industrial statistic prior to 1969. The linkage procedure used for the KVK and EK data has been reported in detail previously (Lynge, 1985).

*Cheminova and Danske gasværkers tjærekompagni* From these companies lists were received of persons known to have been employed in the previous production of MCPA. However, as their completeness could not be checked with external data sources, data from these companies were not considered suitable for inclusion in a cohort analysis.

*Follow-up for death and emigration*

In Denmark, vital status and date of death or emigration is registered in the Central Population Register (CPR) for all persons who have been living in the country since the register was set up on April 1, 1968. Vital status by December 31, 1982 was determined for persons in the cohort with personal identification numbers by a computer based linkage with CPR on January 16, 1984. Persons without personal identification numbers were traced through municipality population registers, the national death index, parish registers and the immigration authority. A total of 30 persons (0.7%) could not be traced and are consequently excluded from the analysis.

*Registration of cancer cases*

A population-based cancer registration was commenced in Denmark in 1943, and patients alive on April 1, 1968 are registered with personal identification numbers. For cohort members with identification numbers notified cancer cases during the period 1943–1982 were identified by linkage with the cancer register on January 16, 1984.

For cohort members without identification numbers notified cancer cases were identified by manual check of lists of cancer patients of equivalent sex and date of birth.

In grouping of the cancer cases advantage was taken of the fact that specific codes have been used for lymphosarcomas and reticulosarcomas and for all other soft tissue sarcomas during the entire registration period in the Danish Cancer Registry. From 1943–1977 notified cases were coded according to a modified version of ICD-7 (Clemmesen, 1974) and from 1978 and onwards according to ICD-O (WHO, 1976). In tabulation STS located in organs are grouped together with STS located in connective tissue.

*Calculation of expected number of cancer cases*

For each individual person years at risk are counted from start of work in the plant (the first years considered were 1947 at KVK and 1951 at EK) until death, emigration or end of follow-up on December 31, 1982. All tumours diagnosed during the individual risk periods are included in the analysis. Expected numbers are based on cancer incidence rates for the Danish population for sex, 5-year age groups and the following calendar periods: 1943–47, 1948–52...1973–77, and 1978–80. Due to the special coding system described above it has been possible to calculate incidence rates for all STS; i.e. in organs and connective tissue. The Monson programme (Monson, 1974) was used for calculation of expected numbers. Ninety-five percent confidence intervals for the relative risks (RR) were calculated assuming that the observed number of cases follows a Poisson distribution and the expected number is constant.

### Results

Table II shows the number of persons included in the analysis and Table III the number of cancer cases. From KVK a total of 3,844 persons is included. Among these are 176 cancer patients with a total of 184 tumours diagnosed in the considered risk period. From EK a total of 615 persons is included, among these are 24 cancer patients each with one tumour. A total of 3,390 males contributing with nearly 50,000 person years at risk, and a total of 1,069 women contributing with nearly 18,000 person years at risk are included in the analysis. In Table IV the persons are tabulated by department in the factories. An employee is counted under a given department if he/she has at least one registered employment period there. The sum of employees in the departments is consequently slightly higher than the total number of employees. A total of 690 males and 250 females had assignment to departments for manufacture and packaging of phenoxy herbicides in the two factories. In the total cohort 59% of the males and 50% of the females had been employed for less than one year. Persons, identified from ATP-records only, had the same proportion of short term employees as the entire cohort.

Table V shows the observed and expected number of cancer cases by diagnostic group for all male and female employees from the two factories

**Table II** Number of persons in cohort

| *Factory* | *Registered number of persons* | *Only work outside plant* | *Untraced* | *Only employed before manufacture of phenoxy herbicide* | *Final number in cohort* |
|---|---|---|---|---|---|
| Kemisk Værk Køge | 3935[a] | 65 | 26 | — | 3844 |
| Esbjerg Kemikaliefabrik | 628[a] | — | 4 | 9[b] | 615 |

[a]One person had been employed at both KVK and EK.
[b]Two persons with employment before and after the manufacture of phenoxy herbicides were excluded from the analysis.

**Table III** Observed cancer cases in risk population

| | *Males* | | | *Females* | | |
|---|---|---|---|---|---|---|
| | *First tumour*[a] | *Second tumour* | *All tumours* | *First tumour*[a] | *Second tumour* | *All tumours* |
| Kemisk Værk Køge | 140 | 7 | 147 | 36 | 1 | 37 |
| Esbjerg Kemikaliefabrik | 12 | — | 12 | 12 | — | 12 |

[a]First tumour after start of employment in factory.

**Table IV** Number of persons and person years at risk by sex, factory and department in factory

| Department in factory | Males | | | | | | Females | | | | | |
|---|---|---|---|---|---|---|---|---|---|---|---|---|
| | *KVK*[a] | | *EK*[b] | | *Total* | | *KVK* | | *EK* | | *Total* | |
| | *N*[c] | *PY*[d] | *N* | *PY* | *N* | *PY* | *N* | *PY* | *N* | *PY* | *N* | *PY* |
| Manufacture and packaging of phenoxy herbicides | 599 | 7226 | 91 | 1151 | 690 | 8377 | 223 | 3912 | 27 | 206 | 250 | 4118 |
| All manual service functions | 934 | 15738 | 54 | 793 | 988 | 16531 | 212 | 3527 | 26 | 471 | 238 | 3998 |
| Manufacture of other substances | 1180 | 18390 | 205 | 2291 | 1385 | 20681 | 118 | 2744 | 164 | 2588 | 282 | 5332 |
| Office | 164 | 2476 | 18 | 379 | 182 | 2855 | 241 | 3407 | 31 | 550 | 272 | 3957 |
| Unspecified | 292 | 3708 | 6 | 117 | 298 | 3825 | 79 | 1148 | 1 | 28 | 80 | 1176 |
| Total | 3021 | 45213 | 369 | 4666 | 3390 | 49879 | 823 | 13835 | 246 | 3789 | 1069 | 17624 |

[a]KVK = Kemish Værk Køge; [b]EK = Esbjerg Kemilkaliefabrik; [c]N = Number of persons; [d]PY = Person years at risk.

**Table V** Observed and expected numbers of cancer cases among all employees from the two factories by diagnostic group. No latency time

| *Diagnosis* | | *Males* | | | *Females* | | |
|---|---|---|---|---|---|---|---|
| *ICD-7* | *Site* | *Obs* | *Exp* | *RR* | *Obs* | *Exp* | *RR* |
| 140–205 | 1. All malignant neoplasms | 159 | 160.61 | 0.99 | 49 | 55.90 | 0.88 |
| 140–195 | 2. Tumours in organs sarcomas excluded | | | | | | |
| 140–145, 147–148 | Buccal cavity and pharynx | 4 | 5.33 | 0.75 | 2 | 0.55 | 3.64 |
| 150 | Oesophagus | 3 | 1.77 | 1.69 | 1 | 0.20 | 5.00 |
| 151 | Stomach | 12 | 9.32 | 1.29 | 1 | 1.47 | 0.68 |
| 153 | Colon | 10 | 9.98 | 1.00 | 1 | 3.51 | 0.28 |
| 154 | Rectum | 14 | 9.15 | 1.53 | 2 | 1.99 | 1.01 |
| 155–156 | Liver | 3 | 3.14 | 0.96 | — | 0.97 | — |
| 157 | Pancreas | 3 | 5.12 | 0.59 | — | 1.13 | — |
| 162 | Lung | 38 | 31.80 | 1.19 | 6 | 2.71 | 2.21 |
| 170 | Breast | — | — | — | 13 | 13.92 | 0.93 |
| 171 | Cervix uteri | — | — | — | 9 | 7.19 | 1.25 |
| 172 | Corpus uteri | — | — | — | 2 | 3.00 | 0.67 |
| 175 | Ovary | — | — | — | 2 | 3.56 | 0.56 |
| 177 | Prostate | 9 | 10.86 | 0.83 | — | — | — |
| 180 | Kidney | 3 | 4.98 | 0.60 | — | 1.09 | — |
| 181 | Bladder | 11 | 13.12 | 0.84 | — | 1.12 | — |
| 190–191 | Skin | 14 | 21.35 | 0.66 | 3 | 5.80 | 0.52 |
| 193 | Brain | 4 | 5.50 | 0.73 | 2 | 1.69 | 1.18 |
| 197[a] | 3. Soft tissue sarcomas | 5 | 1.84 | 2.72 | — | 0.75 | — |
| 196 | 4. Bone | — | 0.45 | — | — | 0.10 | — |
| 198, 200–202[b] | 5. Malignant lymphomas | 7 | 5.37 | 1.30 | 1 | 1.21 | 0.83 |
| 204 | 6. Leukaemia | 5 | 4.51 | 1.11 | 2 | 0.96 | 2.08 |

[a]ICD-7 197: Malignant neoplasms of connective tissue, *and* all sarcomas in organs.
[b]ICD-7 202.0: Brill-symmers' disease *not* included.

together. For males a total of 159 cancer cases was observed *versus* a total of 160.61 expected cases. The highest observed RR for males is found for STS with 5 observed cases *versus* 1.84 expected; RR=2.72, CI95 0.88–6.34. For female employees a total of 37 cancer cases are observed in contrast to 44.44 expected.

In order to take into consideration the possibility that a carcinogenic effect of phenoxy herbicide could show up only after a certain latency time the tabulations presented above have been repeated taking a 10 year latency period into account. For all male employees at the two factories tabulation with a 10 year latency from start of employment shows 105 observed cancer cases in contrast to 108.87 expected. In this tabulation only 4 observed cases of STS *versus* 1.09 expected cases represents a statistical significant excess risk; RR=3.67, CI95 1.0–9.39. When a 10 year latency period is considered for all female employees a total of 34 cancer cases was observed *versus* 38.09 expected. None of the RRs for females are statistically different from unity.

Table VI shows the observed and expected number of cancer cases by diagnostic groups for male and female employees with assignment to departments for manufacture and packaging of phenoxy herbicides. An excess risk of lung cancer is observed among males with 11 observed cases *versus* 5.33 expected; RR 2.06, CI95 1.03–3.69. An equivalent tabulation with a 10 year latency period taken into account shows a total of 22 observed cancer cases among males in the phenoxy herbicide departments *versus* 16.83 expected cases, and a total of 5 cases among females *versus* 9.52 expected. For single diagnostic groups tabulations considered for a 10 year latency period are equivalent to the results shown in Table VI.

As employment registrations from April 1, 1964 and onwards are based on ATP these are known to be complete. A total of 553 males had assignment to departments for manufacture and packaging of phenoxy herbicides at the two plants after this date and a total of 19 cancer cases is observed in this group compared with 17.60 expected. Four cases were located in the rectum where 0.98 were expected; RR=4.08, CI95 1.11–10.45. A total of 169 females had been employed in the phenoxy herbicide department from April 1, 1964 and onwards and this group shows 7 observed cancer cases *versus* 7.53 expected. A significantly excess risk was observed for cancer of the cervix uteri with 4 observed cases compared with 0.85 expected; RR=4.71, CI95 1.28–12.05.

**Table VI** Observed and expected number of cancer cases among employees from the two factories in manufacture and packing of phenoxy herbicides by diagnostic group. No latency time. (Only diagnostic groups with observed cases)

| *Diagnosis* | | *Males* | | | *Females* | | |
|---|---|---|---|---|---|---|---|
| *ICD-7* | *Site* | *Obs* | *Exp* | *RR* | *Obs* | *Exp* | *RR* |
| 140–205 | 1. All malignant neoplasms | 28 | 26.64 | 1.05 | 13 | 15.02 | 0.87 |
| 140–195 | 2. Tumours in organs sarcomas excluded | | | | | | |
| 140–145, 147–148 | Buccal cavity and pharynx | — | 0.87 | — | 1 | 0.14 | 7.05 |
| 150 | Oesophagus | 1 | 0.29 | 3.45 | — | 0.05 | — |
| 151 | Stomach | 2 | 1.47 | 1.36 | — | 0.37 | — |
| 154 | Rectum | 4 | 1.49 | 2.68 | — | 0.54 | — |
| 162 | Lung | 11 | 5.33 | 2.06 | 1 | 0.78 | 1.28 |
| 170 | Breast | — | — | — | 2 | 3.76 | 0.53 |
| 171 | Cervix uteri | — | — | — | 5 | 1.82 | 2.75 |
| 175 | Ovary | — | — | — | 1 | 0.98 | 1.02 |
| 177 | Prostate | 1 | 1.81 | 0.55 | — | — | — |
| 190–191 | Skin | 4 | 3.56 | 1.12 | 1 | 1.56 | 0.64 |
| 197[a] | 3. Soft tissue sarcomas | 1 | 0.30 | 3.33 | — | 0.20 | — |
| 204 | 6. Leukaemia | 1 | 0.74 | 1.35 | 1 | 0.25 | 4.00 |

[a]ICD-7 197: Malignant neoplasms of connective tissue, *and* all sarcomas in organs.

Tables VII and VIII show the observed and expected number of cases among males by department for the two diagnostic groups of main interest on this study. Table VII shows that one of the STS cases in the KVK cohort occurred among men with assignment to departments for manufacture and packaging of phenoxy herbicide, 3 cases occurred among men with assignment to manual service functions, and one case among men with assignment to departments for manufacture of other substances. Table VIII shows that none of the seven ML cases among KVK male employees occurred in the phenoxy herbicide department. Six cases occurred among male employees in manufacture of other substances, mainly pigments, representing a statistical excess risk.

In Table IX the 5 STS patients are listed with diagnoses as reported to the Cancer Registry and employment records in the dataset. The 5 cases represent 5 different tumour types. The patients were between 27 and 64 years at time of diagnosis. Their employment started between 1947 and 1969. Three patients had fairly short employment periods of 3 months, 3 months and half a month. One patient was not among the employees reported on company cards but identified as previously employed at KVK through the pension scheme data (ATP) only. The patients were diagnosed between 5

**Table VII** Observed and expected cases of soft tissue sarcoma among men by department

| | *No latency time* | | | | | | | | | *10 years latency time* | | | | | | | | |
|---|---|---|---|---|---|---|---|---|---|---|---|---|---|---|---|---|---|---|
| | *KVK* | | | *EK* | | | *Total* | | | *KVK* | | | *EK* | | | *Total* | | |
| *Department in factory* | *obs* | *exp* | *RR* | *obs* | *exp* | *RR* | *obs* | *exp* | *RR* | *obs* | *exp* | *RR* | *obs* | *exp* | *RR* | *obs* | *exp* | *RR* |
| Manufacture and packaging of phenoxy herbicides | 1 | 0.26 | 3.91 | — | 0.04 | — | 1 | 0.30 | 3.33 | 1 | 0.14 | 7.16 | — | 0.02 | — | 1 | 0.16 | 6.25 |
| All manual service functions | 3 | 0.58 | 5.19[a] | — | 0.02 | — | 3 | 0.60 | 5.00[c] | 2 | 0.37 | 5.46 | — | 0.02 | — | 2 | 0.39 | 5.13 |
| Manufacture of other substances | 1 | 0.73 | 1.38 | — | 0.07 | — | 1 | 0.80 | 1.25 | 1 | 0.44 | 2.28 | — | 0.03 | — | 1 | 0.47 | 2.13 |
| Office | — | 0.09 | — | — | 0.02 | — | — | 0.11 | — | — | 0.05 | — | — | 0.01 | — | — | 0.06 | — |
| Unspecified | — | 0.11 | — | — | — | — | — | 0.11 | — | — | 0.05 | — | — | 0.00 | — | — | 0.05 | — |
| Total | 5 | 1.68 | 2.98[b] | — | 0.16 | — | 5 | 1.84 | 2.72[d] | 4 | 1.00 | 4.01[e] | — | 0.09 | — | 4 | 1.09 | 3.67[f] |

[a]$CI_{95}$: 1.07–15.12 (poisson); [b]$CI_{95}$: 0.96–6.95 (poisson); [c]$CI_{95}$: 1.03–14.62 (poisson); [d]$CI_{95}$: 0.88–6.34 (poisson); [e]$CI_{95}$: 1.09–10.24 (poisson); [f]$CI_{95}$: 1.00–9.39 (poisson).

**Table VIII** Observed and expected cases of malignant lymphoma among men by department

| | *No latency time* | | | | | | | | | *10 years latency time* | | | | | | | | |
|---|---|---|---|---|---|---|---|---|---|---|---|---|---|---|---|---|---|---|
| | *KVK* | | | *EK* | | | *Total* | | | *KVK* | | | *EK* | | | *Total* | | |
| *Department in factory* | *obs* | *exp* | *RR* | *obs* | *exp* | *RR* | *obs* | *exp* | *RR* | *obs* | *exp* | *RR* | *obs* | *exp* | *RR* | *obs* | *exp* | *RR* |
| Manufacture and packaging of phenoxy herbicides | — | 0.77 | — | — | 0.13 | — | — | 0.90 | — | — | 0.40 | — | — | 0.06 | — | — | 0.46 | — |
| All manual service functions | 1 | 1.68 | 0.59 | — | 0.07 | — | 1 | 1.75 | 0.57 | 1 | 1.03 | 0.97 | — | 0.04 | — | 1 | 1.07 | 0.93 |
| Manufacture of other substances | 6 | 2.07 | 2.91[a] | — | 0.23 | — | 6 | 2.30 | 2.61 | 3 | 1.21 | 2.47 | — | 0.10 | — | 3 | 1.31 | 2.29 |
| Office | — | 0.26 | — | — | 0.05 | — | — | 0.31 | — | — | 0.15 | — | — | 0.03 | — | — | 0.18 | — |
| Unspecified | — | 0.36 | — | — | 0.01 | — | — | 0.37 | — | — | 0.15 | — | — | 0.01 | — | — | 0.16 | — |
| Total | 7 | 4.89 | 1.43 | — | 0.48 | — | 7 | 5.37 | 1.30 | 4 | 2.80 | 1.43 | — | 0.24 | — | 4 | 3.04 | 1.32 |

[a]$CI_{95}$: 1.06–6.31 (poisson).

**Table IX** Soft tissue sarcoma cases

| *Year of birth* | *Cancer registry diagnosis* | *Year of diag- nosis* | *Age at diag- nosis* | *Start of employ- ment at KVK* | *Duration of employ- ment at KVK in months* | *Department at KVK* | *Years from start of employment to diagnosis* | *Final diagnosis in hospital files* | *Cause of death ICD-8* |
|---|---|---|---|---|---|---|---|---|---|
| 1912 | Leiomyo- sarcoma, prostate | 1977 | 64 | 1959 | 30 | Shipping | 17 | Leiomyosarcoma; prostate, wall of bladder or vessel | Malignant neoplasm of prostate (185.x) |
| 1920[b] | Dermatofibro- sarcoma, back | 1968 | 47 | 1st 1947[a] 2nd 1954 | 90 | Shipping | 21 | Haemangiopericytoma; back | Malignant neoplasm of unspecified localization (195.9) |
| 1925[c] | Mesenchymal tumour, larynx | 1982 | 56 | 1956 | 0.5 | Pigment milling | 26 | Leiomyosarcoma; larynx | Alive Jan. 1, 1984 |
| 1946[d] | Fibro- sarcoma, lower limb | 1974 | 27 | 1969 | 3 | Shipping | 5 | Neurofibrosarcoma; lower limb | Malignant neoplasm of connective tissue in lower limbs (171.3) |
| 1948[e] | Sarcoma, retro- peritoneum | 1982 | 34 | 1968 | 3 | Phenoxy herbicide packaging | 14 | Mesenchymal tumour (possible liposar- coma, retroperito- neum | Alive Jan 1, 1984 |

[a]Employment at KVK before 1947 not included. [b]Second primary tumour 1968: Cancer registry diagnosis: haemangiopericytoma back. Final diagnosis in hospital files: The second diagnosis dates back to a review of slides from the first tumour when lung metastasis occurred in 1974. [c]First primary tumour 1951: Cancer Registry diagnosis: lymphosarcoma groin. Final diagnosis in hospital files; possible lymphogranulomatosis. [d]Possible neurofibromatosis. Mother died from neurofibrosarcoma at a young age. [e]Person known from ATP-data.

and 26 years since start of employment at KVK. Hospital files were consulted in order to make sure that the 5 STS cases were correctly reported to the Cancer Registry. All 5 cases were confirmed. Furthermore, the hospital files revealed that the lymphosarcoma reported to the Cancer Registry in 1951 for the patient born in 1925 was never confirmed. The patient born in 1946 probably came from a family with neurofibromatosis. By January 1, 1984 3 of the 5 patients were dead. Only one of the registered causes of death indicated that the malignant tumour was a sarcoma.

### Discussion

The indication from the Swedish studies of the human carcinogenicity of phenoxy herbicides has been followed up in further epidemiological studies. Two additional case-control studies were carried out in Sweden. A study of colon cancer patients was set up in order to control for a potential recall bias in the studies of patients with STS and ML. Colon cancer was not expected to relate to phenoxy herbicide exposure, and the study showed a non-significant RR of 1.3, CI95 0.6–2.8 (Hardell, 1981). Patients with nasal and nasopharyngeal cancer were studied in order to detect a possible risk from inhalation during spraying. This study showed a non-significant RR of 2.1, CI95 0.9–4.7, in men exposed to phenoxy herbicides (Hardell *et al.*, 1982). A New Zealand case-control study covered patients with cancer in connective tissues. Cancer controls were used and the study showed a non-significant RR of 1.6, CI90 0.8–3.2, (Smith *et al.*, 1983).

A cohort study of 1,911 Finnish sprayers showed less cancer deaths than expected (Riihimaeki *et al.*, 1982); and no cases of STS (expected 0.1) or ML (expected 0.5) were observed (Riihimaeki *et al.*, 1983). Among 348 men who sprayed along Swedish railway tracts an excess number of cancer deaths was observed, especially for stomach cancer (Axelson *et al.*, 1980). The cancer mortality was not increased among 145 Swedish forestry workers exposed to phenoxy herbicides, but an excess risk was observed among 16 foremen (Hogsted & Westerlund, 1980). Three follow-up studies have been undertaken of small groups of workers exposed to 2,3,7,8-TCDD during accidents in the manufacture of 2,4,5-trichlorophenol; at BASF AG, German Federal Republic (Thiess *et al.*, 1982), at Monsanto, Nitro, West Virginia, USA (Zack & Suskind, 1980), and at Dow Chemical, USA (Cook *et al.*, 1980). Taken together, the three studies show a RR of 1.29, CI95 0.78–2.01, for overall cancer mortality. In the German study 3 cases of stomach cancer were observed *versus* 0.70 expected (Thiess *et al.*, 1982). No cancer cases were observed among men remaining in the company's employment 10 years after an accident during the manufacture of 2,4,5-trichlorophenol at Coalite, UK (May, 1982). A follow-up study from Dow Chemical, USA, covered 204 men employed in formation of 2,4,5-T; one cancer death was observed *versus* 3.6 expected (Ott *et al.*, 1980). A mortality study of 884 men from the entire Monsanto plant, Nitro, West Virginia, showed an RR for overall cancer mortality of 1.13, CI95 0.79–1.57. Among 163 decedents, 58 had worked in areas of 2,4,5-trichlorophenol or 2,4,5-T production; increased PMRs for lung and bladder cancer were seen both in this group and among decendents from other parts of the plant (Zack & Gaffey, 1983).

As referred to in the introduction three deaths from STS were observed in the US cohorts of workers exposed to 2,3,7,8-TCDD during the manufacture of 2,4,5-trichlorophenol or 2,4,5-T (Honchar & Halperin, 1981). Additionally, one live cohort member was reported to suffer from STS (Cook, 1981). Later 3 cases of STS were reported among workers in 2,4,5-T manufacturing plants (Moses & Selikoff 1981; Johnson *et al.*, 1981). A review of employment records and tissue specimens for the 7 cases suggested that only 2 of the 4 cases identified on studied cohorts were STS; whereas the 3 cases outside cohorts were all confirmed as STS, but for these patients company records showed no specific assignment to 2,4,5-trichlorophenol or 2,4,5-T departments (Fingerhut *et al.*, 1983).

Among 584 cancer cases (excluding non-melanoma of the skin) reported to the Agent Orange Registry, USA, 117 cases were malignant lymphomas; a significantly higher proportion than in the equivalent SEER-data (Young *et al.*, in press). One thousand two hundred and forty-seven Ranch Handers who sprayed Agent Orange in Vietnam in 1961–71 have so far not experienced a higher cancer mortality than an equivalent group of US pilots from the Vietnam War (United States Airforce, 1983). Vietnamese studies have established suggestive evidence of an association between wartime herbicide exposure and primary liver cancer (Westing, 1984).

The observation in the present cohort study of five STS cases in contrast to 1.84 expected among males employed at KVK and EK supports the Swedish observation of an excess risk of STS following exposure to the D and M phenoxy herbicides, However, it is possible to question this conclusion from several points of view.

First, one patient probably had a hereditary predisposition for development neurofibrosarcoma. However, hereditary predisposed cancer cases also contribute to the standard rates, and there is no reason why chemical exposures should not increase the risk of STS in subjects who are already genetically predisposed. The patient is excluded from the calculation when a 10 year latency period is taken into account.

Second, one patient was notified to the Cancer Registry with a lymphosarcoma in the right groin before employment at KVK. Hospital files showed that this diagnosis was never confirmed. The patient had been locally irradiated for his suspected malignancy with a small dose; his second malignancy developed in the larynx. It is unlikely that the larynx dose of the patient has been of any significance as doses received in distant organs are considerably smaller than that of the irradiated site (Stovall, 1983). Here too, it is relevant to point out that cancer cases developed in patients who have previously received radiation therapy contribute to the standard rates.

Third, only one of the patients was assigned to the departments for manufacture and packaging of phenoxy herbicides. It does not seem reasonable, however, to consider the other patients as unexposed. KVK is located with a limited area which is still today marked by the phenoxy herbicide production. Three patients had been employed in the shipping department. In principle only sealed goods are handled here but medical certificates with diagnoses from the 1950s show sick leaves among employees due to cauterizations caused by exposure to other substances in this department. Regarding the possible exposure of manual service workers it is important to take into consideration that the predominant production of the 1960s and 1970s was the spray dried MCPA-sodium salt. If the calculation is limited to males with assignment to departments for manufacture and packaging of phenoxy herbicides, manual service functions and unspecified, a total of four STS cases are observed in contrast to 1.01 expected RR = 3.96, CI96 1.08–10.14.

Fourth, the employment periods for three of the patients are very short; 3 months. 3 months and half a month, respectively. This, however, accords with the observation from the Swedish case-control studies. In the study from Northern Sweden 5 out of 13 exposed cases had worked with phenoxy herbicides for 3 months or less (Hardell & Sandstrøm, 1979). One of the patients in the cohort ran a small farm beside his work at the plant, making additional exposure during spraying possible.

Fifth, the STS risk is only observed among males. Among females only a total of 0.75 cases is expected, and 22% of the female person years at risk derives from office employees in contrast to only 6% among males. The majority of the female office employees had worked at the KVK Copenhagen office.

Sixth, the patients could have been exposed to 2,4,5-T. Such exposure is unlikely due to the limited amount of 2,4,5-T processed at KVK. Only one of the patients had been employed during the years 1951–52 when a total of 5 tons 2,4,5-trichlorophenol was produced.

Last, occupational mortality data from the UK indicate a slight, social class gradient in the mortality from cancer of the connective tissue (ICD 8, 171) (OPCS, 1978); equivalent data are not available from Denmark. As the cohort members mainly belong to social classes III, IV and V one may ask whether the observed RR of 2.72 for STS in this study reflects risk factors related to social class than to the work place. However, standardization for social class based on the UK data would only cause a marginal decrease in the observed RR, and the relevance of the UK data is difficult to evaluate as tumours of the connective tissue (ICD 8, 171) only constitute about one fourth of the incident STS cases in Denmark.

Seven ML cases were observed among males in contrast to 5.37 expected, giving of RR = 1.30, C195 0.52–2.69. The Swedish study of male ML patients showed a RR of 4.8, C195 2.9–8.1 (Hardell *et al.*, 1981). Furthermore, 6 of the 7 ML cases in the present study occurred among males assigned to the department for manufacture of pigments at KVK. Thus, concerning ML the results in this cohort study do not support the Swedish observation.

The total number of cancer cases among the 3,021 males in the cohort is equivalent to the expected number. For the 1,069 females the total number of cancer cases is below the expected number, with the deficit coming from KVK. The same results are obtained after :xclusion of non-melanoma skin cancers for which there might be a diagnostic bias. Furthermore, the total number of cancer cases among the 690 males with assignment to departments for manufacture and packaging of phenoxy herbicide is also close to the expected, whereas it is below the expected for the 250 females within these departments. The overall cancer incidence is consequently not increased either among all employees at the two plants or among employees with assignment to the phenoxy herbicide departments. However, it is important in evaluation of these results to consider that according to data on the work force reported to the industrial statistics the KVK cohort is not entirely complete for the first 10–15 years after the manufacture of phenoxy herbicides was commenced in 1947.

For single diagnostic groups other than STS and ML the study has shown an excess risk for lung cancer; RR = 2.06, C195 1.03–3.69, and for rectum cancer; RR = 4.08, C195 1.11–10.45, for males with assignment to departments for manufacture and packaging of phenoxy herbicides, and an excess risk of cervical cancer; RR=4.71, CI95 1.28–12.05 for females with assignment to these departments. These observations derive from tabulations with 17 diagnostic groups for males and 20 for females and may consequently be due to chance. The excess lung cancer risk is seen among males at both KVK and EK. Both plants are located near provincial towns and workers were previously recruited mainly from the countryside, where the tobacco consumption was relatively low in the 1950s (Lindhardt, 1960). Seven of the patients had worked in the 1960s and 1970s where the spray dried MCPA-sodium salt dominated. Although phenoxy herbicide was predominant other substances were produced as well (see Figure 1). However, none of these is known to be associated with an excess lung cancer risk. Three KVK workers had previous assignment to the pigment department where zinc chromate was formerly produced as one of the many pigments. Exposure to zinc chromate is associated with an excess lung cancer risk (Langaard & Vigander, 1983). Three patients were notified to the Cancer Registry with occupations recorded in the national statistic with excess lung cancer risks; baker, butcher and brewery worker (Danmarks Statistik, 1979). Previously, a non-significant excess lung cancer risk had been observed among white males employed in manufacture of 2,4,5-T for more than one year (Zack & Gaffey, 1983). An excess lung cancer risk was also observed among pesticide workers exposed to a variety of pesticides including 2,4-D and MCPA (Barthel, 1976). Based on the data presented here it is not possible to draw a conclusion concerning the lung cancer risk following exposure to phenoxy herbicides.

The excess risk of rectal cancer observed among males assignmed to the phenoxy herbicide departments is surprising as the Swedish case-control study showed a non-significant relative risk for colon cancer of 1.3 (Hardell, 1981) and the two diseases are aetiologically closely related. The present study is the first including females exposed to phenoxy herbicides. An excess risk of cervical cancer is observed, and a total of 5 cervical cancer patients had previous assignment to the phenoxy herbicide packaging department at KVK in the early 1960s. It is not possible, based on the available data, to draw a conclusion as to the aetiology of this excess risk of cervical cancer.

It was the repeated observation in the Swedish case-control studies of a RR of 5–6 for STS following exposure to phenoxy herbicides that caused concern in agencies for occupational health and safety about the possible carcinogenity in humans of phenoxy herbicides. The present cohort study has shown that several potential biases have to be taken into account when the Swedish observations are tested in other settings. Among the five STS patients in the present study one was identified as cohort member based on the pension scheme data (ATP) only. For the three dead patients only one of the recorded causes of death indicates a STS. It is consequently necessary for a retesting of the Swedish observations on a cohort design to have both a complete registration of exposed persons and a population based cancer register with data on both topography and morphology. Even in Denmark it has been difficult to fulfil the first of these requirements, and a continuous follow-up of the complete cohort of persons employed in the two factories after 1964 is desirable.

Vibeke Syppli Enrum and Lis Mikkelsen assisted with the data collection. Claus Danø Møller and Niels Christensen of the Danish Cancer Registry and Debbie White of the National Cancer Institute, USA, assisted with the data processing. The study was supported by the Danish Work Environment Foundation (1982–40).

### References

AXELSON, O., SUNDELL, L., ANDERSSON, K., EDLING, C. & KLING, H. (1980). Herbicide exposure and tumor mortality. *Scand. J. Work Environ. Hlth*, **6**, 73.

BARTHEL, E. (1976). High incidence of lung cancer in persons with chronic professional exposure to pesticides in agriculture. *Z. Erkrank. Atm-Org.*, **146**, 266.

BOVEY, R.W. & YOUNG, A.L. (1980). *The Science of 2,4,5-T and Associated Phenoxy Herbicides*. New York.

CLEMMESEN, J. (1974). Statistical studies in the aetiology of malignant neoplasms. IV. Denmark 1943–67. *Acta Pathol. Microbiol Scand. (Suppl.)*, **247**, 19.

COLE, P. (1980). Direct testimony before the *Environmental Protection Agency of the United States of America*. October 1980.

COOK, R.R., TOWNSEND, J.O., OTT, M.G. & SILVERSTEIN, L.G. (1980). Mortality experience of employees exposed to 2,3,7,8-tetrachlorodibenzo-p-dioxin (TCDD). *J. Occ. Med.*, **22**, 530.

COOK, R.R. (1981). Dioxin, chloracne and soft-tissue sarcoma. *Lancet*, **i**, 618.

DANMARKS STATISTIK. (1979). *Occupational Mortality 1970–75* (In Danish). Copenhagen.

ERIKSSON, M., HARDELL, L., BERG, N.O., MOELLER, T. & AXELSON, O. (1981). Soft tissue sarcomas and exposure to chemical substances: A case-referent study. *Br. J. Ind. Med.*, **38**, 27.

FINGERHUT, M.A., HALPERIN, W.E., HONCHER, P.A., SMITH, A.B., GROTH, D.H. & RUSSEL, W.O. (1984). An evaluation of reports of dioxin exposure and soft tissue sarcoma pathology among chemical workers in the United States. *Scand. J. Work. Environ. Health*, **10**, 299.

HARDELL, L. (1977). Malignant mesenchymal tumors and exposure to phenoxy herbicides – a clinical observation. (In Swedish). *Laekartidn.*, **74**, 2753.

HARDELL, L. (1979). Malignant lymphoma of histiocytic type and exposure to phenoxy-acetic acids or chlorophenols. *Lancet*, **i**, 55.

HARDELL, L. & SANDSTRØM, A. (1979). A case-control study: Soft-tissue sarcomas and exposure to phenoxyacetic acids or chlorophenols. *Br. J. Cancer*, **39**, 711.

HARDELL, L. (1981). On the relation of soft tissue sarcoma, malignant lymphoma and colon cancer to phenoxy acids, chlorophenols and other agents. *Scand. J. Work. Environ. Hlth*, **7**, 119.

HARDELL, L., ERIKSSON, M., LENNER, P. & LUNDGREN, E. (1981). Malignant lymphoma and exposure to chemicals, especially organic solvents, chlorophenols and phenoxy acids. A case-control study. *Br. J. Cancer*, **43**, 169.

HARDELL, L., JOHANSSON, B. & AXELSON, O. (1982). Epidemiological study of nasal and nasopharyngeal cancer and their relation to phenoxy acid or chlorophenol exposure. *Am J. Ind. Med.*, **3**, 247.

HOGSTED, C. & WESTERLUND, B. (1980). Cohort study of mortality among forestry workers with and without exposure for phenoxy herbicides (In Swedish). *Laektidn.*, **77**, 1828.

HONCHAR, P.A. & HALPERIN, W.E. (1981). 2,4,5-T, trichlorophenol and soft-tissue sarcoma. *Lancet*, **i**, 268.

JOHNSON, F.E., KUGLER, N.A. & BROWN, S.M. (1981). Soft tissue sarcomas and chlorinated phenols. *Lancet*, **i**, 1370.

LANGAARD, S. & VIGANDER, T. (1983). Occurrence of lung cancer in workers producing chromium pigments. *Br. J. Ind. Med.*, **40**, 71.

LINDHARDT, M. (1960). The sickness survey of Denmark 1951–54. Copenhagen.

LOOS, M.A. (1975). Phenoxyalkanoic acids. In *Herbicides, Chemistry, Degradation and Mode of Action*. Vol. I. (Eds. Kearney & Kaufmann). Dekker, New York.

LYNGE, E. (1985). Background and design of a Danish cohort study of workers in phenoxy herbicides manufacture. *Am. J. Ind. Med.* (In press).

MAY, G. (1982). Tetrachlorodibenzodioxin: A survey of subjects ten years after exposure. *Br. J. Ind. Med.*, **39**, 128.

MONSON, R.R. (1974). Analysis of relative survival and proportional mortality. *Comp. Biomed. Res.*, **7**, 325.

MOSES, M. & SELIKOFF, F.J. (1981). Soft-tissue sarcomas, phenoxy herbicides and chlorinated phenols. *Lancet*, **i**, 40.

OPCS. (1978). Occupational mortality. Decennial supplement 1970–72 for England and Wales. London.

OTT, M.G., HOLDER, B.B. & OLSON, R.D. (1980). A mortality analysis of employees engaged in the manufacture of 2,4,5-trichlorophenoxyacetic acid. *J. Occ. Med.*, **22**, 47.

RIIHIMAEKI, V., SISKO, A. & HERNBERG, S. (1982). Mortality of 2,4-dichlorophenoxy-acetic acid and 2,4,5-trichlorophenoxyacetic acid herbicide applicators in Finland. *Scand. J. Work Environ. Hlth*, **8**, 37.

RIIHIMAEKI, V., ASP, S., PUKKALA, E. & HERNBERG, S. (1983). Mortality and cancer morbidity among chlorinated phenocyacid applicators in Finland. *Chemophere*, **12**, 779.

SMITH, A.H., FISHER, D.O., GILES, H.J. & PEARCE, N. (1983). The New Zealand soft tissue sarcoma study. Interview findings concerning phenoxyacetic acid exposure. *Chemophere*, **12**, 565.

STOVALL, M. (1983). Organ doses from radiotherapy of cancer of the uterine cervix. In *Second Cancer in Relation to Radiation Treatment for Cervical Cancer*. (Eds. Day & Boice) IARC Scientific Publications no. 52; Lyon.

SØRUP, P. (1982). Chemistry. In *Chlorophenols and Phenoxy-herbicides*. (Ed. Arbejdstilsynet). (In Danish): Copenhagen.

THIESS, A.M., FRENTZEL-BEYME, R. & LINK, R. (1982). Mortality study of persons exposed to dioxin in a trichlorophenol-process accident that occurred in the BASF AG on November 17, 1983. *Am. J. Ind. Med.*, **3**, 179.

UNITED STATES AIR FORCE. PROJECT RANCH HAND II. (1983). *An Epidemiologic Investigation of Health Effects in Air Force Personnel Following Exposure to Herbicides*. Texas, USAF.

WESTING, A.H. (ed). (1984). Herbicides in war. The long-term ecological and human consequences. SIPRI: London.

WHO. (1976). ICD-O. *International Classification of Diseases for Oncology*. Geneva: WHO.

YOUNG, A.L., FLICKER, M.R., KANG, H.K. & SHEPERD, B.M. Health surveillance of Vietnam veterans claiming agent orange exposure. In *Chlorinated Dioxins and Dibenzofurans in the Total Environment-II*. Symposium proceedings. *Am. Chem. Soc.*, (In press).

ZACK, J.A. & SUSKIND, R. (1980). The mortality experience of workers exposed to tetrachlorodibenzodioxin in a trichlorophenol process accident. *J. Occ. Med.*, **22**, 11.

ZACK, J.A. & GAFFEY, W.R. (1983). A mortality study of workers employed at the Monsanto company plant in Nitro, West Virginia. *Environ. Sci. Res.*, **26**, 575.